# A Systematic Review of High Quality Diagnostic Tests for Chagas Disease

**DOI:** 10.1371/journal.pntd.0001881

**Published:** 2012-11-08

**Authors:** Anna M. Afonso, Mark H. Ebell, Rick L. Tarleton

**Affiliations:** 1 Department of Epidemiology and Biostatistics, University of Georgia, Athens, Georgia, United States of America; 2 Center for Tropical and Emerging Global Diseases and Department of Cellular Biology, University of Georgia, Athens, Georgia, United States of America; National Institutes of Health, United States of America

## Abstract

**Background:**

There is significant heterogeneity in reported sensitivities and specificities of diagnostic serological assays for Chagas disease, as might be expected from studies that vary widely according to setting, research design, antigens employed, and reference standard. The purpose of this study is to summarize the reported accuracy of serological assays and to identify sources of heterogeneity including quality of research design. To avoid associated spectrum bias, our analysis was limited to cohort studies.

**Methods:**

We completed a search of PubMed, a bibliographic review of potentially relevant articles, and a review of articles identified by a study author involved in this area of research. Studies were limited to prospective cohort studies of adults published since 1985. Measures of diagnostic accuracy were pooled using a Der Simonian Laird Random Effects Model. A subgroup analysis and meta regression were employed to identify sources of heterogeneity. The QUADAS tool was used to assess quality of included studies and Begg's funnel plot was used to assess publication bias.

**Results:**

Eighteen studies and 61 assays were included in the final analysis. Significant heterogeneity was found in all pre-determined subgroups. Overall sensitivity was 90% (95% CI: 89%–91%) and overall specificity was 98% (95% CI: 98%–98%).

**Conclusion:**

Sensitivity and specificity of serological assays for the diagnosis of Chagas disease appear less accurate than previously thought. Suggestions to improve the accuracy of reporting include the enrollment of patients in a prospective manner, double blinding, and providing an explicit method of addressing subjects that have an indeterminate diagnosis by either the reference standard or index test.

## Author Summary

Chagas disease, an infectious disease endemic to Latin America, is caused by the protozoan parasite *Trypanosoma cruz*i. *T. cruzi* can be transmitted through blood transfusions, organ transplants, or from mother to fetus, although it is most commonly transmitted through insect vectors. Infections can remain silent for many years before manifesting as potentially fatal damage to the cardiac and/or digestive system. Diagnosis of Chagas disease during its chronic asymptomatic phase is crucial to preventing future infections with *T. cruzi* and is often performed using serological tests that detect antibodies in the blood. Because there is currently no gold standard for serological diagnostic tests, multiple forms of serologic testing are often used in conjunction. The purpose of this study was to compare reports on the accuracy of serological tests. After limiting studies by certain criteria, the authors found a lower estimate of accuracy than has previously been reported in the literature and suggest quality improvements that can be made to standardize future reports.

## Introduction

Chagas disease, or American Trypanosomiasis, is caused by the parasite *Trypanosoma cruzi*. The World Health Organization estimates that approximately 10 million individuals are currently infected with *T. cruzi* and are at risk for developing cardiac or gut pathology normally associated with chronic Chagas disease [1]. *T. cruzi* is transmitted to humans by infected triatomine bugs that infest housing, take blood meals from the inhabitants, and then defecate, leaving the infective metacyclic stages of *T. cruzi* to be scratched into wounds or mucosal sites. Although Chagas disease was once confined to the Americas, primarily Latin America, migration from endemic countries has led to the appearance of Chagas disease in non-endemic regions as well [1]. In both endemic and non-endemic settings, transmission of *T. cruzi* is also possible through blood transfusion, tissue transplantation, and congenitally. Control programs and improvements in housing have led to a reduction in the incidence of disease in Latin America, but screening blood donors and diagnosing chronic, often asymptomatic patients, remains a major challenge.

*T. cruzi* infection is generally controlled by a highly effective immune response but is rarely completely cleared, resulting in a persistent, but low level infection. Early in the infection with *T. cruzi*, parasites may be detected in the bloodstream either by direct observation of blood or by various culture techniques. Unfortunately, infection at this early stage often goes undetected because symptoms are nonspecific or absent. Once the immune response to *T. cruzi* is established, parasite detection is very difficult and diagnosis of the infection is based largely upon the detection of anti-*T. cruzi* antibodies by serological techniques. Conventional serological tests include primarily immunofluorescence assays (IFA), enzyme-linked immunosorbent assays (ELISA) and indirect hemagglutination assays (IHA).

Because there is currently no single reference standard test, the World Health Organization (WHO) recommends that diagnosis of an individual utilize two conventional tests based on different principles and detecting different antigens [2]. In the case of ambiguous or discordant results, a third technique should be used [2]. The goal of this study is to summarize the evidence on the accuracy of diagnostic tests for Chagas disease from high quality diagnostic test studies. Previous research has found that use of a case-control design overestimated the diagnostic odds ratio (DOR) by 3-fold compared to studies employing a cohort design [3]. In order to more accurately assess the sensitivity and specificity of serological assays used to screen patients for Chagas disease, we limited our review to studies that prospectively enrolled patients using a cohort study design.

## Methods

### Inclusion and Exclusion Criteria

Criteria for inclusion in this systematic review were that the study 1) be available in English, Spanish, Portuguese, or German; 2) be published since 1985; 3) use human subjects rather than model organisms; 4) include a minimum of 50 samples; 4) prospectively enroll patients without knowledge of *T. cruzi* infection status using a cohort design; 5) examine a serologic assay based on measuring antibody levels in the blood (e.g. ELISA, IHA, IFA, immunochromatographic assay, complement fixation), rather than a PCR assay, urine analysis, or saliva test; 6) provide enough information to determine sensitivity and specificity of a serologic assay compared with a reference standard of some kind; 7) enroll primarily adolescents or adult patients (12 years or older). The year 1985 was chosen as a cutoff to capture the time period during which conventional tests of IFA, IHA, and ELISA came into wide use in Latin America [4]. Studies involving exclusively immunodeficient adults, patients with HIV or TB, children, infants, neonates, or pregnant women were excluded. We excluded any study designed to evaluate serologic tests as a means to assess cure of Chagas disease, as the purpose of this systematic review is to evaluate the performance of diagnostic tests in patients with unknown disease status. Case-control studies that identified a group of well characterized cases and well characterized normal controls, often from different sources, were excluded because this is an important methodological limitation [3]. Data for patients with the acute stage of *T. cruzi* infection were excluded from the analysis, given that these data represented only a small proportion of all studies and provided extremely heterogeneous results. Furthermore, it has been shown that the reactivity of IgG antibodies to various antigens varies between patients with the acute and chronic form of Chagas disease [5].

### Search Strategy

We used several strategies to identify relevant articles, including searching PubMed with multiple search strategies, reviewing bibliographic citations from both included and excluded articles, and reviewing studies known to be relevant by one of the study authors, an expert in the field of Chagas diagnostics. The following strategy was used in PubMed/Medline to identify studies providing a quantitative evaluation of diagnostic tests for Chagas disease: *(“chagas disease/diagnosis”[MeSH Major Topic]) AND (“sensitivity and specificity”[MeSH Terms] OR “likelihood ratio”)*. We identified 156 articles, of which 121 were identified as potentially relevant by one or more authors based on a review of the abstracts. These articles were then assessed for inclusion criteria based on a review of the full text of each article.

Medline (PubMed) was also searched for previous systematic reviews on the diagnosis of Chagas disease. The following strategy was used: *(“diagnosis”[Subheading] OR “diagnosis”[All Fields] OR “diagnosis”[MeSH Terms]) AND Chagas[All Fields]) AND systematic[sb] = (Diagnosis Chagas) AND systematic[sb])*. This search identified 12 studies, one of which was considered potentially relevant based on a review of abstracts [6]. This systematic review, however, was excluded after a review of the full text.

Eight full text articles were provided by a study author (RLT) as being potentially relevant. In addition to reviewing these articles, we reviewed the bibliographies of all included studies, as well as bibliographies of studies excluded based on the criteria of having a case control rather than cohort design. We also performed a search of LILACS to capture articles that may have been missed by other methods. Our search strategy was to use the search term “Chagas,” limit study type to cohort studies and limit clinical aspect to “diagnosis.” This identified 15 studies, of which none met our study criteria. We also performed this search using “Trypanosoma cruzi” and “T. cruzi” as the search term. We found no additional studies.

### Data Abstraction

Abstracts and full texts were evaluated for inclusion criteria by three blinded reviewers; one assessed all articles and each of the other two reviewers assessed about half of the articles. Information regarding the characteristics of each study and the data needed to create a contingency (“2×2”) table comparing each index test with its reference standard were abstracted into a worksheet by two reviewers in a blinded fashion. If a study reported sera that were indeterminate by the index test, we considered indeterminate results as a positive index test, and incorporated them into the assessment of accuracy of the test whenever possible. The methodological quality of each included study was also independently assessed by two reviewers using the QUADAS tool (Quality Assessment of Diagnostic Accuracy Studies) [7]. Differences between reviewers were resolved through consensus discussion.

### Statistical Analysis

Sensitivity, specificity, positive likelihood ratios (LR+), and negative likelihood ratios (LR-) with 95% CI's were calculated for each test and then pooled using a Der Simonian Laird Random Effects Model. For studies with cells in the 2×2 table containing a value of zero, 0.5 was added to all cells to avoid division by zero. Heterogeneity of pooled sensitivity and specificity was estimated with the I^2^ statistic, where a value for I^2^ of 0 indicates perfect homogeneity (all of the variance is within study) whereas a value of 1.0 indicates perfect heterogeneity (all of the variance is between studies). The area under a summary ROC curve for pooled results was also calculated using a Der Simonian Laird Random Effects Model. Because there was little variation in specificity estimates, a bivariate method was not used. Calculations were performed with MetaDisC ver 1.4 (Madrid, Spain).

Sources of heterogeneity were assessed by meta-regression using the metareg command in Stata version 11.0 (College Station, TX) under a random effects model. The independent variable was the log of the diagnostic odds ratio (DOR), which compares the odds of having a positive test result in those with T. cruzi compared to the odds of having a positive test result in those uninfected with T. cruzi [8]. It is calculated as (TP/FP)/(FN/TN) [9]. Within study variance was estimated by the standard error of the ln(DOR), calculated as the square root of [(1/TP)+(1/FP)+(1/FN)+(1/TP)] [9]. A random effects meta regression does not assume that all variability exist within a study but also takes into account between study variability in the model [10]. Between study variance was estimated by a restricted likelihood method using an iterative procedure. A fixed effects meta-regression was not performed because this model assumes that all the heterogeneity can be explained by the posited covariates [11] while a random effects model allows for unexplained heterogeneity. Publication bias and small study bias were assessed with a Begg funnel plot using the metabias command in Stata Version 11.0 (College Station, TX).

## Results

### Included Studies

Eighteen studies met the inclusion criteria. Thirteen studies were included based on a search of Medline, one more was included from the personal file of a study author, and four more were included based on a review of citations of included studies and non-included case-control studies. The included cohort studies were published between 1988 and 2010. All but one included study took place in Central America or South America. The one remaining study took place in Switzerland, although participants were Latin American immigrants. Seven studies (39%) were conducted with blood bank donors. The remaining studies came from “field studies”, population surveys, or other settings.

Twelve studies (67%) used subjects with unknown symptoms, while the remaining studies classified participants as asymptomatic or with clinical evidence of Chagas disease. Participants consisted mainly of adults with a mean age in the mid twenties; 8 studies did not report any information on the age of subjects, while 10 did not report any information regarding the sex distribution of participants. The characteristics of each study included in this analysis are outlined in Table S1.

### Study Quality

**Figure 1:**
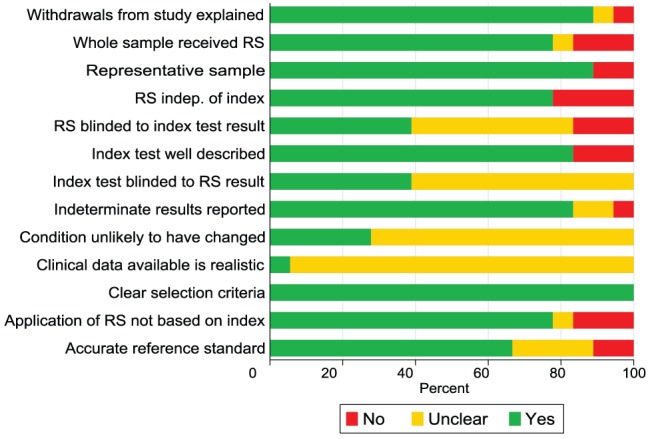
Overall percentage of studies that met QUADAS guidelines. * RS =  Reference standard.

**Figure 2:**
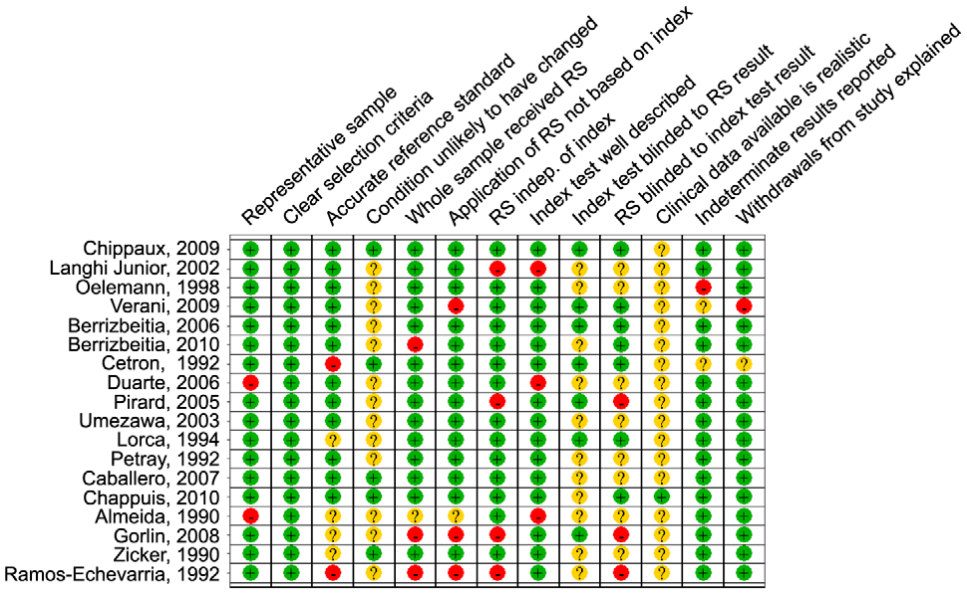
Quality assessment of individual studies using QUADAS tool. * RS =  Reference standard.

A QUADAS score was created by assigning one point to all QUADAS criteria answered positively, 0.5 of a point for studies in which it was unclear whether a criteria was met, and zero points when a study clearly did not meet a QUADAS criteria. Figure 1 summarizes the percentage of studies meeting each of the QUADAS quality criteria, and Figure 2 provides an overview of the quality criteria for each study.

### Index Tests

Of the 61 tests assessed, 44 were ELISA, 3 were immunofluorescence assays, 7 were indirect hemagglutination assays, 4 were immunochromatographic assays, one was a chemilimunescence assay, one was a Dot-ELISA, and one was a multiple antigen binding assay (MABA). Antigens used in each of the assays are described in Table S2. Twenty six of sixty one assays (43%) did not report the antigen used in an assay; among those that did, 15 assays used a form of recombinant antigen while 28 used a fixed or whole form of the parasite. Of the 61 assays included in this review, 36 were commercial assays. Details regarding type of commercial assay used are also described in Table S2.

We were unable to use information regarding cutoffs to complete a threshold analysis because of the limited number of studies that reported cutoffs used. In fact, 39 (64%) did not report the exact cutoff or even methodology used to determine the cutoff (i.e. 2.5 SD's above the mean of uninfected patients).

### Reference Standards

Because there is no single widely accepted reference standard test for assessing Chagas disease, included studies used a wide variety of methods to classify true positives and true negatives. Four of eighteen studies used a single test as the reference standard. Because the reference standard was either positive or negative, there was no issue of having discordant test results for the reference standard. However, a single reference standard test is unlikely to correctly classify the index test [2]. Four studies used a reference standard of 2 or more out of 3 serological tests being positive as a true positive; three studies considered 2 out of 2 tests as a true positive and everything else as a true negative; and two studies considered 3 out of 3 positive tests as a true positive. Two studies used latent class analysis, which uses the results of index tests to approximate the true unobserved disease state [12] to identify true positives and negatives. Three studies used another method or did not describe the method used for determining true positives and negatives. Details of the reference standard methodology are summarized in Table S1. Only 4 of 18 studies reported the number of samples that were discordant by the combination of serological assays used to determine the true status of a sample.

### Diagnostic Accuracy

**Figure 3:**
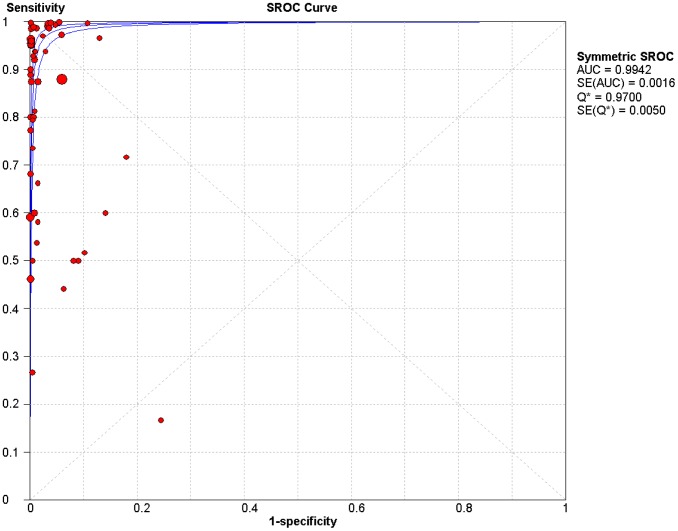
Summary receiver operating characteristic curve with 95% CI for all tests. Each circle represents the sensitivity and specificity of an individual assay. The size of the circles reflects the number of patients in a study. Area under the curve = 0.99.

The area under the summary ROC curve (Figure 3) for all assays was 0.99 (SE = 0.002). In the summary ROC curve, it appears that there may be a positive correlation between sensitivity and (1-specificity), suggesting that some of the heterogeneity in sensitivity and specificity estimates is due to the use of differing cutoffs. However, when we performed a Spearman rank correlation, there was little evidence of a threshold effect (Spearman correlation coefficient = 0.04, p = 0.76). An apparent outlier in the summary ROC curve is a study [13] that reported a low sensitivity for nearly all assays. This reduced accuracy is likely due to the fact that each assay included only one recombinant antigen, while most conventional assays use a combination of multiple antigens or whole parasite extracts. Removing this outlier only increased the sensitivity slightly but did not change the specificity (Table 1).

**Table 1:** Pooled results with an assessment of heterogeneity.

	No assays	No studies	N	Sens (95% CI)	I^2^	Range	Spec (95% CI)	I^2^	Range	LR+ (95% CI)	LR− (95% CI)	AUC	SE(AUC)
*All studies*	61	18	18, 458	0.90 (0.89,0.91)	96%	0.17–1.00	0.98 (0.98,0.98)	97%	0.76–1.0	73 (51, 104)	0.09 (0.06, 0.15)	0.99	0.002
*Outlier study removed*	57	17	18,210	0.93 (0.93–0.94)	94%	0.17–1.00	0.98 (0.98–0.98)	97%	0.76–1.00	94 (65,136)	0.08 (0.05, 0.13)	1.0	0.001
*ELISA*	45	15	14, 882	0.90 (0.89,0.91)	96%	0.44–1.00	0.98 (0.98, 0.98)	97%	0.82–1.00	82 (54, 126)	0.09 (0.05, 0.15)	0.99	0.002
*Not ELISA*	16	9	3,576	0.90 (0.88,0.92)	96%	0.17–1.00	0.98 (0.97, 0.98)	98%	0.76–1.00	57 (26, 125)	0.10 (0.03, 0.36)	0.99	0.004
*Commercial Tests*	36	12	7,930	0.95 (0.94,0.95)	94%	0.17–1.00	0.99 (0.99, 0.99)	97%	0.76–1.00	113 (68, 190)	0.07 (0.03,0.18)	1.00	0.001
*Non Commercial Tests*	25	11	10,528	0.81 (0.80, 0.83)	96%	0.44–1.00	0.97 (0.97,0.98)	97%	0.82–1.00	44 (26, 74)	0.17 (0.10,0.28)	1.00	0.005
*Reference standard*													
*Single test positive*	16	4	1,815	0.75 (0.73, 0.78)	94%	0.17–1.00	0.93 (0.92, 0.94)	96%	0.76–1.00	19 (9.1, 38)	0.29 (0.17, 0.48)	0.96	0.018
*2/3 positive*	10	4	2,717	0.84 (0.80, 0.87)	93%	0.46–1.00	1.00 (1.00, 1.00)	94%	0.89–1.00	180 (35, 924)	0.14 (0.06, 0.32)	1.00	0.002
*2/2 positive*	6	3	8,246	0.97 (0.96, 0.98)	95%	0.88–1.00	0.96 (0.95, 0.96)	97%	0.94–1.00	80 (23, 274)	0.02 (0.007, 0.081)	1.00	0.002
*3/3 positive*	4	2	1,408	0.42 (0.35, 0.49)	88%	0.27–1.00	0.99 (0.99, 1.0)	76%	0.99–1.00	86 (31, 243)	0.53 (0.31, 0.89)	0.98	0.013
*Latent Class Analysis*	10	2	2,351	0.98 (0.97, 0.99)	74%	0.60–1.00	0.99 (0.99, 0.99)	97%	0.87–1.00	52 (23, 115)	0.02 (0.007, 0.08)	1.00	0.0013
*Other*	15	3	1,921	0.97 (0.95, 0.98)	82%	0.60–1.00	1.00 (1.00, 1.00)	80%	0.96–1.00	293 (131,654)	0.09 (0.04, 0.24)	1.00	0.0008
*Blinding Reported*	32	7	5,391	0.84 (0.82–0.85)	96%	0.27–1.00	0.99 (0.99–0.99)	95%	0.82–1.00	96 (53–175)	0.11 (0.07–0.19)	0.99	0.0022
*Blinding not Reported*	29	11	13,067	0.95 (0.94–0.96)	93%	0.17–1.00	0.97 (0.97–0.97)	97%	0.76–1.00	57 (35, 93)	0.08 (0.04–0.17)	0.99	0.0025
*Blinding Reported and Adequate Reference standard*	7	2	2,369	0.53 (0.46–0.59)	94%	0.27–1.00	1.00 (1.00–1.00)	40%	0.99–1.00	344 (79–1488)	0.31 (0.15–0.63)	1.00	0.0001
*Blinding and Adequate Reference standard not present*	54	16	16, 089	0.92 (0.91–0.93)	95%	0.17–1.00	0.98 (0.97–0.98)	97%	0.76–1.00	56 (40–79)	0.09 (0.05–1.42)	0.99	0.0022
*Rec with outlier removed*	11	8	2,724	0.90 (0.88–0.91)	98%	0.27–1.00	0.99 (0.98–0.99)	92%	0.89–1.00	94 (33, 264)	0.05 (0.01–0.20)	1.00	0.002
*Recombinant*	15	9	2,972	0.82 (0.80, 0.84)	98%	0.27–1.00	0.98 (0.97, 0.98)	95%	0.82–1.00	37 (17, 84)	0.08 (0.02, 0.27)	0.99	0.007
*Not recombinant*	28	9	12,805	0.95 (0.94, 0.96)	92%	0.50–1.00	0.98 (0.98, 0.98)	97%	0.91–1.00	117 (65, 214)	0.07 (0.04, 0.14)	1.00	0.001
*Blood bank*	35	7	7,295	0.96 (0.94, 0.97)	82%	0.17–1.00	0.99 (0.99, 0.99)	97%	0.76–1.00	136 (74,249)	0.09 (0.04, 0.19)	1.0	0.002
*Not blood bank*	26	11	11,163	0.88 (0.87, 0.89)	98%	0.27–1.00	0.96 (0.96, 0.96)	94%	0.82–1.00	33 (21, 54)	0.09 (0.05, 0.18)	0.99	0.004
*Unknown only*	41	12	15,422	0.91 (0.89,0.92)	94%	0.27–1.0	0.99 (0.99, 0.99)	96%	0.94–1.0	194 (112, 338)	0.10 (0.06, 0.19)	1.0	0.001
*Other*	20	6	3,036	0.90 (0.88,0.91)	97%	0.17–1.0	0.94 (0.93, 0.94)	96%	0.76–1.0	16 (9.9, 26)	0.07 (0.03, 0.17)	0.98	0.008
*Discordants reported*	8	4	2,754	0.94 (0.93, 0.95)	99%	0.27–1.00	0.98 (0.98, 0.98)	95%	0.90–1.00	62 (25, 153)	0.03 (0.003, 0.28)	1.0	0.002
*Discordants not reported*	53	14	15,704	0.88 (0.87, 0.89)	94%	0.17–1.00	0.98 (0.98, 0.98)	97%	0.76–1.00	76 (51, 113)	0.12 (0.076, 0.18)	0.99	0.002
*QUADAS high (>10)*	35	9	11,707	0.80 (0.79,0.82)	91%	0.44–1.00	0.98 (0.98,0.99)	97%	0.82–1.00	127 (71, 228)	0.17 (0.12, 0.25)	0.99	0.002
*QUADAS low (0–10)*	26	9	6,751	0.96 (0.95, 0.96)	97%	0.17–1.00	0.98 (0.97, 0.98)	97%	0.76–100	42 (26, 67)	0.04 (0.01, 0.14)	0.99	0.002

**Figure 4:**
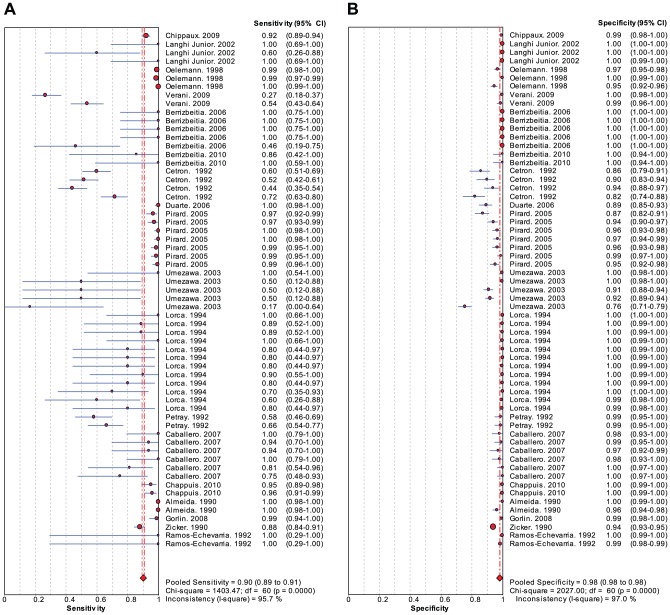
Forest plot of sensitivity and specificity for all studies.

A Forest plot (Figure 4) of sensitivity and specificity estimates for each study (with 95% CI's) reveals that specificity is high and consistent between studies, while estimates of sensitivity vary more widely. While we report summary estimates for sensitivity and specificity, they should be interpreted with great caution given the significant heterogeneity and design limitations of the included studies.

Table 1 shows pooled results for several predetermined subgroup analyses. Studies with a QUADAS score above 10 (better designed studies) had a lower sensitivity than less well designed (80% [95% CI:79–82%] vs 96%[95%CI:95–96%], p = 0.07). ELISA tests and non-ELISA tests were similarly sensitive and specific. Commercial tests were more sensitive than non-commercial tests (95% [95%CI: 94–95%] vs 81% [95% CI: 80–83%],p = 0.08) but had similar specificity (99% [95%CI:99–55%] vs 97% [95%CI:97–98%], p = 0.57). Sensitivity and specificity were higher in studies conducted with blood bank samples compared to tests evaluated in field studies (96% [95%CI: 94–97%] vs 88% [95%CI: 87%–89%], p = 0.24 sensitivity and 99% [95%CI:99–99%] vs 96%[95%CI:96–96%], p = 0.44 specificity). In the stratification by reference standard used, sensitivity was lowest among studies requiring 3/3 positive tests as a reference standard (42% [95%CI:35–49%]). Overall, sensitivity and specificity were low for studies using a single test as a reference standard (75% [95%CI:73–78%]) and 93%[95%CI: 92–94%], respectively).

Results of the metaregression, which examined the independent effect of study design characteristics on the diagnostic odds ratio, are shown in Table 2. Study design characteristics significantly associated with the diagnostic odds ratio included whether an ELISA or other assay was employed (relative diagnostic odds ratio [RDOR] = 4.22), whether a study utilized latent class analysis as the reference standard (RDOR = 90), and whether the index test was blinded to the reference standard (RDOR 0.03). Thus, studies that were blinded reported a lower estimate of diagnostic accuracy, while those using latent class analysis and those using an ELISA assay reported a higher estimate of accuracy. One limitation of this analysis is that the sample size for the metaregression is the number of studies, not the number of patients, and thus there may not be sufficient power to detect other important effects [10].

**Table 2:** Meta regression analysis.

Covariate	Coefficient	P>|z|	RDORφ	95% confidence intervalφ
Discordant Handling Reported vs Discordant Handling Not Reported	2.109573	0.198	8.24472	(0.33–204)
Classification of symptoms is unknown vs Classification of symptoms reported	−0.72771	0.58	0.483012	(0.04–6.36)
Quadas Scoreψ	0.728601	0.058	2.07218	(1.00–4.40)
Elisa assay vs other assay*	1.440274	0.034	4.221852	(1.12–15.96)
Study of blood bank samples vs other setting	1.130894	0.262	3.098425	(0.43–22.34)
Commercial assay vs non commercial assay	0.015853	0.986	1.01598	(0.18–5.62)
Reference standard requires 2/3 positive tests	1.187613	0.318	3.279244	(0.32–33.80)
References standard requires 2/2 positive tests	1.045418	0.523	2.844587	(0.12–70.21)
Reference standard requires 3/3 positive tests	0.563691	0.792	1.757147	(0.03–115)
Reference standard is latent class analysis*	4.506194	0	90.57643	(11.45–716)
Other reference standard*	4.85111	0	127.8823	(11.03–1482.27)
Blinding vs no blinding*	−3.41986	0.007	0.032717	(0.003–0.40)
Blinding and acceptable reference standard vs no blinding and lack of acceptable reference standard	2.702167	0.084	14.91201	(0.69–320.77)
Intercept	−2.7737	0.431	0.06243	(0.00–62.35)

Ψ Continuous variable ranging from 6.5 to 12.5.

Φ RDOR and 95% CI's obtained by exponentiating coefficients and respective confidence intervals.

* significant at p<0.05.

**Figure 5:**
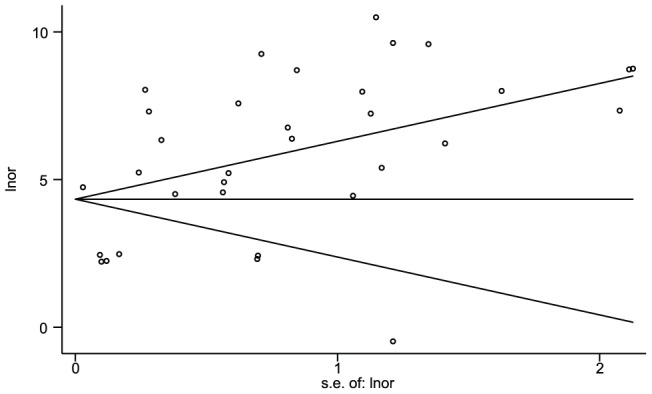
Assessment of publication bias with Begg's funnel plot. The measure of effect is ln(DOR) and the standard error of the ln(DOR) is the measure of variance. Note relative deficit of smaller studies showing lower accuracy in the lower right quadrant of the figure.

Publication bias is the tendency of smaller studies with null results to go unpublished while studies showing an effect are more likely to appear in the literature [10]. To assess this effect, we built a funnel plot (Figure 5) of the log of the diagnostic odds ratio against the standard error of the log of the diagnostic odds ratio, an indicator for sample size. Each open circle in the funnel plot represents an individual assay and the line in the center represents the summary diagnostic odds ratio. A gap in the plot of missing values below the summary DOR line is an indication of publication bias.

In a regression of the standardized effect estimates against their precisions, we found a positive association between smaller studies and a higher reported DOR (slope = 4.47, 95% CI: 3.6–5.3, t = 10.47, p<0.001). The intercept of the fitted line can be interpreted as a measure of bias [14] (Intercept: −0.58; 95% CI: −3.71, 2.63; t = −0.35; p = 0.732). A gap in the expected funnel shape of the scatter plot indicates that there may be publication bias, with a lack of smaller studies reporting negative findings.

## Discussion

Previous studies have reported extremely high values for the sensitivity and specificity of serologic tests for Chagas disease. For example, the recent systematic review by Brasil and colleagues found summary estimates of sensitivity and specific for ELISA of 97.7% (96.7%–98.5%) and 97.5% (88.5%–99.5%) respectively. Summary estimates for commercial ELISA were a pooled sensitivity of 99.3% (97.9%–99.9%) and a pooled specificity of 97.5% (88.5%–99.5%). Despite these supposedly high sensitivity/specificity levels for tests, a number of groups recommend or advise the use of multiple tests for accurate diagnosis [6], [15] suggesting that many experts are still skeptical of the high accuracy of individual tests reported in the literature.

As can be seen in the QUADAS table (Figure 2), there are several biases inherent in the manner in which the reference standard was applied. In some studies, sera with results borderline by the index test were excluded [16], [17], [18], thereby inflating estimates of sensitivity and specificity. Several studies based the decision to apply the reference standard on the results of the index test, incorporated the index test into the reference standard, or did not apply a uniform reference standard to all sera in the study. In one study, only patients with a positive index test and a random selection of negative samples received the reference standard [19]. In other studies RIPA was only applied as part of the reference standard if the index or reference test was positive, while those initially negative either did not receive this test or only a random sample received the RIPA test [17], [20]. In another study, only samples positive by the index ELISA test were submitted to another ELISA test, and only those positive by both ELISA tests were submitted to a Western Blot. Those reactive by all three tests were considered positive. However, only those positive by the index test were even eligible to be considered positive; any false negatives misclassified by the index test received no other verification and would be assumed to be true negatives. One study [21] repeated tests for which the index test and reference standard test were discordant. However, when the index test is applied in clinical settings, there will be no such gold standard to monitor the veracity of a test. In other studies [16], [22], initially positive or indeterminate samples were evaluated in duplicate and considered positive only when a repeated test was positive. Therefore, it is important to acknowledge that the reported accuracy in many studies is not that of the index test alone, but rather the accuracy of the entire testing strategy that was implemented.

An important source of bias in these studies is dealing with samples that are discordant by the reference standard. In four of the eighteen studies, discordant samples were reported to have been discarded from sensitivity and specificity calculations (as sensitivity and specificity of a test cannot be calculated with knowledge of the true disease status). This would tend to inflate estimates of sensitivity and specificity. Although eight studies reported the manner in which they handled samples discordant by the reference standard, only four reported the number of samples which were discordant by the reference standard. In these four cases, the percentage of discordant samples out of the total was 7% (23/335) [17], 4% (40/1025) [18], 4% (17/398) [23], and 0.3% (3/999) [24]. In a study by Zicker and colleagues, results for IFA and HA were reported for all sera without specifying a clear reference standard. We chose to consider all specimens with both serology tests positive as a true positive and all other sera as true negatives. This study reported a much lower sensitivity (88%; 95%CI: 84%–91%) and somewhat lower specificity (94%; 95%CI: 93%–95%) than the pooled estimates (97%; 95% CI: 96%–98% and 96%; 95%CI: 95%–96%) for the other studies in that subgroup (those using a reference standard of 2/2 positive tests). All other studies in this subgroup reported a sensitivity greater than 95% and specificity greater than 95%. Because we created the reference standard groupings, we are certain that there were no discordant samples discarded, most likely accounting for the approximately 10% discrepancy in sensitivity estimate. This raises the question of whether other studies had a significant number of unreported discordant samples that would inflate the sensitivity of their tests.

Study design characteristics associated with biased estimates of sensitivity and specificity included different reference tests for those with a positive and negative index tests, failure to blind or mask, and case-control instead of cohort design [3]. In our study, only five of eighteen studies reported that the results of the diagnostic tests and reference tests were both blinded to the other, and only thirteen of eighteen studies applied a uniform reference standard to all or a random sample of subjects.

Our findings are consistent with these general principles of diagnostic study design, as we found increased estimates of sensitivity and specificity in studies with lower quality (QUADAS<10 points) and in unblinded studies. This highlights the serious limitations in the existing literature. We are concerned that the sensitivity of current tests is lower than generally reported in lower quality studies, leading to the potential for underdiagnosis. This is a particular concern when these tests are used as a screening test for blood bank samples. The use of case control design continues to be the most widespread method to evaluate diagnostic tests for *T. cruzi* infection. Based on this analysis, we recommend that future studies use a prospective cohort design with clear reporting, masking, and an appropriate reference standard to provide a more accurate estimate of the diagnostic accuracy of tests for Chagas disease. Our results also suggest that better tests are still needed to assure the safety of transfusions and to improve the public health of countries where the disease is endemic.

## Supporting Information

Table S1 **Characteristics of studies and reference standards.** * The above abbreviations are defined as follows: TESA: Trypomastigote excreted-secreted antigen; ELISA: Enzyme-linked immunosorbent assay; EIA: Enzyme immunoassay; IHA: Indirect hemagglutination test; iIF: Indirect immunofluorescence assay; IFA: Immunofluorescence Assay; Ch: Chagasic; Non-Ch: Non Chagasic; IND: Indeterminate; RIPA: Radioimmunoprecipitation assay; OD: Optical density; DHA: Direct-hemagglutination test; WB: Western blot; SD: Standard deviation; CSP: Chagas Stat-Pak; sens: sensitivity; spec: specificity; LCA: Latent class analysis.
(DOCX)

Table S2 **Description of studies and assays.** ELISA = enzyme linked immunosorbent assay; IHA = Indirect Hemagglutination; IIF =  Indirect Immunofluorescence; IFA =  Immunofluorescence Assay.
(DOCX)

Appendix S1 **Criteria used for QUADAS scoring.** Because we limited our analysis to cohort studies, the majority of studies were considered to have a spectrum of patients representative of those that would be tested in clinical practice. Studies using two or more serologic tests or Latent Class Analysis as a reference standard were considered to have an adequate reference standard. Studies failing to report screening methodology used for both non-Chagasic and Chagasic groups were scored as unclear. Studies using only one reference standard were considered to have an inadequate reference standard. For the majority of studies, the timeline for implementing the index test and reference standard was unclear, making it impossible to determine whether the condition under study was likely to have changed between the index and reference standard. In most cases, the whole sample received the reference standard and the application of the reference standard was not based on the index test. This is more likely to be the case among cohort studies as the disease status of patients is unknown when index tests and the reference standard is applied. Blinding was reported in few studies, and it was generally unclear what information was available to the readers of diagnostic assays.
(DOC)

Figure S1 **PRISMA flow diagram.** This flow diagram maps the identification of records identified, included, and excluded at different phases of the systematic review.
(DOC)

Checklist S1 **PRISMA checklist.** Preferred Reporting Items for Systematic Reviews and Meta-Analyses (PRISM) is an evidenced-based checklist that clearly demonstrates essential items reported in this systematic review.
(DOC)
