# Supplementary material for: A Systematic Review of High Quality Diagnostic Tests for Chagas Disease
Source: PLoS Negl Trop Dis. 2012 Nov 8;6(11):e1881. doi: 10.1371/journal.pntd.0001881 (PMC3493394; doi:10.1371/journal.pntd.0001881)
Supplement: Table S1 — Characteristics of studies and reference standards. * The above abbreviations are defined as follows: TESA: Trypomastigote excreted-secreted antigen; ELISA: Enzyme-linked immunosorbent assay; EIA: Enzyme immunoassay; IHA: Indirect hemagglutination test; iIF: Indirect immunofluorescence assay; IFA: Immunofluorescence Assay; Ch: Chagasic; Non-Ch: Non Chagasic; IND: Indeterminate; RIPA: Radioimmunoprecipitation assay; OD: Optical density; DHA: Direct-hemagglutination test; WB: Western blot; SD: Standard deviation; CSP: Chagas Stat-Pak; sens: sensitivity; spec: specificity; LCA: Latent class analysis. (DOCX) [file pntd.0001881.s001.docx]

**Table S1. Characteristics of studies and reference standards.**

| **Author, year** | **Age** | **Sex**  **(% male)** | **Country** | **Population description** | **Reference standard** | **Discordant handling** | **Comment** |
| --- | --- | --- | --- | --- | --- | --- | --- |
| 1. Chippaux, 2009 | Mean: 25.4 | 47.20% | Bolivia | 995; 482 asymptomatic chagasic; 513 asymptomatic non-chagasic | Commercial ELISA test | Tests giving indeterminate results (those with 2 SD's around cutoff) were repeated. If the index (CSP) and reference standard (ELISA) disagreed, both tests were repeated. | Randomly selected inhabitants of a rural area highly endemic for T. cruzi |
| 2. Langhi Junior, 2002 | NA | NA | Brazil | 1951; 10 chagasic, 1941 non-chagasic* | Latent Class Analysis using information from EIA, IHA, and iIF | The IHA and EIA index tests were repeated if initially reactive and only considered positive if repeated tests were positive | Blood donors from a non-endemic area without risk history for T. cruzi infection (screened by questionnaire) |
| 3. Oelemann, 1998 | NA | NA | Brazil | 1025; 520 chagasic, 505 non chagasic*,40 discordants | Ch: 2/2 Positive serology tests (IFA and ELISA); Non-Ch: 2/2 Negative serology tests (IFA and ELISA); IND: Discrepant results (IFA and ELISA) | 40 samples had discrepant results by the reference standard (excluded from sens/spec), 9 had discrepant (repeatedly indeterminate) results with Abbot ELISA, 11 had discrepant results with BioELISACRUZI kit, and 0 had inconclusive results with BIOZIMA Chagas kit. Results indeterminate by index tests were also omitted from the sensitivity and specificity calculation. | Sera were obtained from patients and healthy residents in various regions of Brazil where the cardiac form of Chagas disease is prevalent, where the indeterminate form of Chagas disease is prevalent, and where Chagas disease is emergent. |
| 4. Verani, 2009 | Mean (range): 16 (2-68) | 48.00% | Peru | 335; 97 chagasic, 238 non chagasic*, 23 discordants | Ch:ELISA, IFA and RIPA positive; Non-Ch: ELISA and IFA negative (RIPA negative in 46) | Specimens with inconclusive index tests results or discordant reference standard readings were excluded from the analysis. | Participants were recruited in public schools and from the entire population of one hillside shantytown |
| 5. Berrizbeitia, 2006 | Mean (range): 29 (18-58) | 89% | Venezuela | 2028; 13 chagasic, 2025 non chagasic* | Ch: 2+ positive reference standard tests (EIA, IIF, IHA); Non-Ch: Less than 2+ positive reference standard tests (EIA, IIF, IHA); 140 randomly selected + indeterminate (OD 0.3-0.4) were verified as negative. | Unable to determine how discordant samples were handled | Consecutive blood bank samples; Well defined samples (150) and samples from non-traveling Canadians (166) were used as controls |
| 6. Berrizbeitia, 2010 | Mean (range): 23 (8-79) | 48.00% | Venezuela | 66; 7 chagasic, 59 non chagasic* | All sera with OD > 0.4 and 14 randomly selected negatives underwent battery of three tests (IF, IHA, EIA) at NCIL. Ch: 2/3 positive tests was considered a true positive Non-Ch: all others were considered true negative | While 2/3 positive serology assays is considered positive, it is unclear whether the remaining samples were considered negative or whether some samples were discarded. Only a random sample of 14 of the negative assays underwent reference standard testing. Inconclusive tests were not mentioned or addressed. | Residents of rural villages |
| 7. Cetron, 1992 | NA | NA | Brazil | 241; Chagasic: 63 with clinical symptoms, 18 asymptomatic, and 39 with unknown symptoms; Non-Chagasic: 128 with unknown symptoms | IFA | There were no discordant results as only 1 reference standard was used. It appears that no samples were excluded. | Two cohorts: population survey (n=82) and hospitalized pts (n = 166) |
| 8. Duarte, 2006 | Range: 10-80 | NA | Brazil | 398; Chagasic: 99 with clinical symptoms, 63 asymptomatic; Non-Chagasic: 236 asymptomatic, 17 discordants | Ch: At least 2 out of 3 positive tests (IFA,IH, and ELISA); Non-Ch: 3 negative tests (IFA,IH,ELISA); IND: 1 positive and 2 negative tests (IFA,IH,ELISA) | 23 samples were discordant by the reference standard. | . |
| 9. Pirard, 2005 | Mean: 31.1 | 66.50% | Bolivia | 400; 146 asymptomatic chagasic, 254 asymptomatic non-chagasic | Latent class analysis | LCA was used to estimate true positives and negatives. | Random sample of blood donors |
| 10. Umezawa, 2003 | NA | NA |  | 451; 6 asymptomatic chagasic, 445 asymptomatic non-chagasic | TESA blot | It appears that no samples were excluded for having a discordant reference standard (only one test was used) or inconclusive index tests | Blood bank samples excluded because of discrepant results on conventional tests. Inconclusive counted as positive for calculations of se/sp. |
| 11. Lorca, 1994 | NA | NA | Chile | 226; 29 chagasic, 2235 non-chagasic* | IF and immunoperoxidate (unclear if both or either positive) | It appears that no discordant or inconclusive index test results were excluded. | . |
| 12. Petray, 1992 | (8 months-84 yrs); 72/215 under age 10 | 59.00% | Argentina | 215; 74 chagasic, 141 non-chagasic* | Ch: Positive by two serology tests out of three (DHA, iHA, iIF); Non-Ch: Unclear what criteria is but because all samples are accounted for, we can assume samples must be negative by two serology tests out of three (DHA, iHA, iIF) | It appears that 2/3 positive serology tests were considered positive and all others were considered negative. | . |
| 13. Caballero, 2007 | 18-60 | NA | Panama | 120; 16 chagasic, 104 non-chagasic* | TESA Western blot | There were no discordant results because only 1 test was used as reference standard. It also appears that inconclusive results by index test were excluded. Samples with inconclusive results were tested at least twice on different days. | . |
| 14. Chappuis, 2010 | 36 (median) | 17.50% | Switzerland | 999; 125 chagasic, 874 non-chagasic*, 3 discordant samples | Two commercial ELISA tests (Biomerieux and Biokit) positive/negative; if discrepant, sent to reference lab for four serologic tests and final determination | 3 samples excluded because reference standard was non-conclusive | Latin-American immigrants |
| 15. Almeida, 1990 | NA | NA | Brazil | 543: 221 chagasic, 242 non-chagasic, *80 non chagasic with other diseases | Unknown | It appears that no samples were excluded from the original study population. However, it is unclear what the reference standard is for the calculation of sensitivity and specificity. | Positive and negative sera from endemic region; Non-Ch: RF (8), Schisto (15), malaria (12), toxo (10), syphilis (14), Leish (21) |
| 16. Gorlin, 2008 | NA | NA | Bolivia, Columbia, Guatemala, Mexico, Nicaragua | 574: 95 chagasic, 479 non-chagasic* | IFA; RIPA if either index or IFA positive | This is not addressed, but it appears that there were no discordant reference standard results or indeterminate index test results. | High-risk population |
| 17. Zicker, 1990 | NA | NA | Brazil | 6222: 357 chagasic, 5865 non-chagasic* | Ch: IF and HA positive; Non-Ch: IF or HA positive, or both negative | We considered samples with discordant results between IF and IHA as negative. | Population survey of manual workers in urban areas |
| 18. Ramos-Echevarria, 1992 | 18-50 | 86% | Mexico | 1076: 3 chagasic, 1073 non-chagasic* | Ch: Positive by Dot ELISA, ELISA, and WB; Non-Ch: Negative by DOT-ELISA or Negative by ELISA or Negative by WB (Not positive by all three) | Samples initially positive by Dot-ELISA were submitted to ELISA, and if still reactive, they were submitted to WB. Only those that were reactive by all three were considered positive. All others were considered negative. It appears that there were no inconclusive or discordant results, and that they were not excluded from the analysis. | Other disease: Leishmaniasis (50), Tuberculosis(10), Cardiomyopathy (32) |

* The above abbreviations are defined as follows: TESA: Trypomastigote excreted-secreted antigen; ELISA: Enzyme-linked immunosorbent assay; EIA: Enzyme immunoassay; IHA: Indirect hemagglutination test; iIF: Indirect immunofluorescence assay; IFA: Immunofluorescence Assay; Ch: Chagasic; Non-Ch: Non Chagasic; IND: Indeterminate; RIPA: Radioimmunoprecipitation assay; OD: Optical density; DHA: Direct-hemagglutination test; WB: Western blot; SD: Standard deviation; CSP: Chagas Stat-Pak; sens: sensitivity; spec: specificity; LCA: Latent class analysis
