# Supplementary material for: A Systematic Review of High Quality Diagnostic Tests for Chagas Disease
Source: PLoS Negl Trop Dis. 2012 Nov 8;6(11):e1881. doi: 10.1371/journal.pntd.0001881 (PMC3493394; doi:10.1371/journal.pntd.0001881)
Supplement: Table S2 — Description of studies and assays. ELISA = enzyme linked immunosorbent assay; IHA = Indirect Hemagglutination; IIF = Indirect Immunofluorescence; IFA = Immunofluorescence Assay. (DOCX) [file pntd.0001881.s002.docx]

**Table S2. Description of studies and assays.**

| **Study ID** | **Author (year)** | **Test ID** | **Test** | **Antigen** | **ELISA** | **Recombinant** | **Commercial** |
| --- | --- | --- | --- | --- | --- | --- | --- |
| 1 | Chippaux, 2009 | 1 | Immunochromat-ographic assay | Recombinant antigens | N | Y | Chagas Stat Pak; Chembio Diagnostic Systems, Medford, NY, USA |
| 2 | Langhi Junior, 2002 | 2 | ELISA | Purified epimastigote and amastigote antigens | Y | N | Abbott Chagas, Abbott, USA |
|  |  | 3 | IHA | Epimastigote and amastigote antigens | N | N | Biolab Diagnostica, RJ, Brazil |
|  |  | 4 | IIF | epimastigote | N | N | Biolab Diagnostica, RJ, Brazil |
| 3 | Oelemann, 1998 | 5 | ELISA | . | Y | N | Abbott Laboratories; San Paulo, Brazil |
|  |  | 6 | ELISA | . | Y | N | BIOELISACRUZI, Biolab-Meriuex; Rio de Janeiro, Brazil |
|  |  | 7 | ELISA | . | Y | . | BIOZIMA Chagas kit, Polychaco S.A.I.C., Buenos Aires, Argentina |
| 4 | Verani, 2009 | 8 | Immunochroma-tographic assay | Recombinant antigen | N | Y | Chagas Stat Pak; Chembio Diagnostic Systems, Medford, NY, USA |
|  |  | 9 | ELISA | Recombinant antigen | Y | Y | Trypanasoma Detect (InBios International, Seattle, WA-a prototype not yet commercially available) |
| 5 | Berrizbeitia, 2006 | 10 | ELISA | Fixed epimastigote | Y | N | . |
|  |  | 11 | ELISA | Fixed typomastigote | Y | N | . |
|  |  | 12 | ELISA | TESA Tulahuen | Y | N | . |
|  |  | 13 | ELISA | TESA Brazil | Y | N | . |
|  |  | 14 | ELISA | . | Y | . | EIA Chagas IgG, Pharmatest |
| 6 | Berrizbeitia, 2010 | 15 | Multi-Antigen Binding Assay | TESA | N | N | . |
|  |  | 16 | ELISA | Fixed epimastigote | Y | N | . |
| 7 | Cetron, 1992 | 17 | ELISA | SA85-1.2 recombinant antigen | Y | Y | . |
|  |  | 18 | ELISA | SA85-1.1 recombinant antigen | Y | Y | . |
|  |  | 19 | ELISA | FL-160 recombinant antigen | Y | Y | . |
|  |  |  | ELISA | Any of 3 positive: SA85-1.2, SA85-1.1 or FL-160 recombinant antigens | Y | Y | . |
| 8 | Duarte, 2006 | 20 | Chemiluminescence | Recombinant antigen | N | Y | Immulite Chagas IgG commercial kit (DPC-MedLab-Brazil) and Immulite system(DPC-MedLab) |
| 9 | Pirard, 2005 | 21 | IHA | . | N | . | Polychaco, Buenos Aires, Argentina |
|  |  | 22 | IHA | . | N | . | Polychaco, Buenos Aires, Argentina |
|  |  | 23 | IFA | Epimastigote | N | N | . |
|  |  | 24 | ELISA | Lyophilized crude antigen | Y | N | . |
|  |  | 25 | ELISA | Crude antigen | Y | N | Gull laboratories |
|  |  | 26 | ELISA | Recombinant antigen | Y | Y | Chagatest, Wiener Lab, Argentina |
|  |  | 27 | ELISA | Recombinant antigen | Y | Y | BIOSChile, Santiago, Chile |
| 10 | Umezawa, 2003 | 28 | ELISA | Mix recombinant protein | Y | Y | . |
|  |  | 29 | ELISA | Epimastigote alkaline extract | Y | N | . |
|  |  | 30 | ELISA | Epi-ELISA | Y | N | . |
|  |  | 31 | IFA | . | N | . | . |
|  |  | 32 | IHA | . | N | . | Immunologic Technology Industry and Commerce |
| 11 | Lorca, 1994 | 33 | ELISA | . | Y | . | Chagatest, Inst Invest. De la Salud |
|  |  | 34 | ELISA | . | Y | N | Ortho Chagas, Prod Profesionales, Argentina |
|  |  | 35 | IHA | . | N | . | Estabilgen Hemo Chagas, Polychaco SA, Argentina |
|  |  | 36 | ELISA | . | Y | . | Abbott Chagas, Abbott, USA |
|  |  | 37 | ELISA | . | Y | . | Chagatest, Inst Invest. De la Salud |
|  |  | 38 | ELISA | . | Y | N | Ortho Chagas, Prod Profesionales, Argentina |
|  |  | 39 | IHA | . | N | . | Estabilgen Hemo Chagas, Polychaco SA, Argentina |
|  |  | 40 | ELISA | . | Y | . | Abbott Chagas, Abbott, USA |
|  |  | 41 | ELISA | . | Y | . | Chagatest, Inst Invest. De la Salud |
|  |  | 42 | ELISA | . | Y | N | Ortho Chagas, Prod Profesionales, Argentina |
|  |  | 43 | IHA | . | N | . | Estabilgen Hemo Chagas, Polychaco SA, Argentina |
|  |  | 44 | ELISA | . | Y | . | Abbott Chagas, Abbott, USA |
| 12 | Petray, 1992 | 45 | ELISA | Circulating antigens (cAg) | Y | N | . |
|  |  | 46 | ELISA | Immune complexes | Y | N | . |
| 13 | Caballero, 2007 | 47 | ELISA | Epimastigote | Y | N | . |
|  |  | 48 | ELISA | . | Y | . | Chagas III, BIOSCHile, Chile |
|  |  | 49 | ELISA | . | Y | N | ELISAcruzi, bioMerieux Brasil |
|  |  | 50 | ELISA | . | Y | . | Chagatek, bioMerieux Brasil |
|  |  | 51 | ELISA | . | Y | Y | Chagatest Rec v3.0, Wiener Laboratories |
|  |  | 52 | ELISA | . | Y | . | Pathozyme Chagas (Omega) |
| 14 | Chappuis, 2010 | 53 | Immunochromatographic assay | Recombinant proteins | N | Y | Chagas Stat Pak; Chembio Diagnostic Systems, Medford, NY, USA |
|  |  | 54 | Immunochromatographic assay | Recombinant proteins | N | Y | Chagas Stat Pak; Chembio Diagnostic Systems, Medford, NY, USA |
| 15 | Almeida, 1990 | 55 | ELISA | CRA+FRA (cytoplasmic repetitive antigen + flagellar repetititve antigen) | Y | Y | . |
|  |  | 56 | ELISA | Cytosolic | Y | N | . |
| 16 | Gorlin, 2008 | 57 | ELISA | . | Y | N | Ortho T. cruzi ELISA Test System (Ortho-Clinical Diagnostics) |
| 17 | Zicker, 1990 | 58 | ELISA | Crude epimastigote | Y | N | . |
| 18 | Ramos-Echevarria, 1992 | 59 | ELISA | Soluble antigen | Y | N | . |
|  |  | 60 | Dot-ELISA | Whole antigen | Y | N | . |

ELISA = enzyme linked immunosorbent assay; IHA = Indirect Hemagglutination; IIF= Indirect Immunofluorescence; IFA= Immunofluorescence Assay
