# Supplementary material for: A Systematic Review of High Quality Diagnostic Tests for Chagas Disease
Source: PLoS Negl Trop Dis. 2012 Nov 8;6(11):e1881. doi: 10.1371/journal.pntd.0001881 (PMC3493394; doi:10.1371/journal.pntd.0001881)
Supplement: Appendix S1 — Criteria used for QUADAS scoring. Because we limited our analysis to cohort studies, the majority of studies were considered to have a spectrum of patients representative of those that would be tested in clinical practice. Studies using two or more serologic tests or Latent Class Analysis as a reference standard were considered to have an adequate reference standard. Studies failing to report screening methodology used for both non-Chagasic and Chagasic groups were scored as unclear. Studies using only one reference standard were considered to have an inadequate reference standard. For the majority of studies, the timeline for implementing the index test and reference standard was unclear, making it impossible to determine whether the condition under study was likely to have changed between the index and reference standard. In most cases, the whole sample received the reference standard and the application of the reference standard was not based on the index test. This is more likely to be the case among cohort studies as the disease status of patients is unknown when index tests and the reference standard is applied. Blinding was reported in few studies, and it was generally unclear what information was available to the readers of diagnostic assays. (DOC) [file pntd.0001881.s003.doc]

Appendix A. Criteria used for QUADAS scoring.

Because we limited our analysis to cohort studies, the majority of studies were considered to have a spectrum of patients representative of those that would be tested in clinical practice. Studies using two or more serologic tests or Latent Class Analysis as a reference standard were considered to have an adequate reference standard. Studies failing to report screening methodology used for both non-Chagasic and Chagasic groups were scored as unclear. Studies using only one reference standard were considered to have an inadequate reference standard. For the majority of studies, the timeline for implementing the index test and reference standard was unclear, making it impossible to determine whether the condition under study was likely to have changed between the index and reference standard. In most cases, the whole sample received the reference standard and the application of the reference standard was not based on the index test. This is more likely to be the case among cohort studies as the disease status of patients is unknown when index tests and the reference standard is applied. Blinding was reported in few studies, and it was generally unclear what information was available to the readers of diagnostic assays.
