# Supplementary material for: A Systematic Review of High Quality Diagnostic Tests for Chagas Disease
Source: PLoS Negl Trop Dis. 2012 Nov 8;6(11):e1881. doi: 10.1371/journal.pntd.0001881 (PMC3493394; doi:10.1371/journal.pntd.0001881)
Supplement: Figure S1 — PRISMA flow diagram. This flow diagram maps the identification of records identified, included, and excluded at different phases of the systematic review. (DOC) [file pntd.0001881.s004.doc]

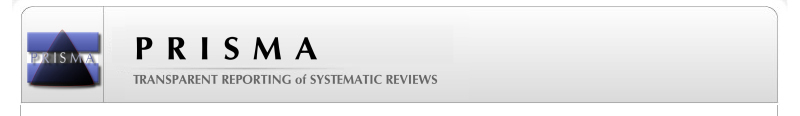
**PRISMA 2009 Flow Diagram**

*Additional records identified through other sources included a bibliographic review of studies considered potentially relevant after a search of pubmed. Duplicates were not monitored during the review process as they were very frequent and thus this value had to be roughly estimated.

**Screening**

**Included**

**Eligibility**

**Identification**

Records excluded
(n = 30 )

Full-text articles excluded

(n =237 )

Not cohort design= 169

Sample size <50 = 21

No serology= 43

No sens/spec data= 74

Not adult (>=18)/ human population=31

Records identified through database searching
(n =156 )

Additional records identified through other sources
(>300* )

Records after duplicates removed
(n =285)

Records screened
(n = 285 )

Full-text articles assessed for eligibility
(n =255)

Studies included in qualitative synthesis
(n =18 )

Studies included in quantitative synthesis (meta-analysis)
(n =18 )
